# Electrodeposition of Ag/ZIF-8-Modified Membrane for Water Remediation

**DOI:** 10.1021/acs.langmuir.2c02947

**Published:** 2023-01-30

**Authors:** Ricky Rodriguez, Miguel S. Palma, Deepali Bhandari, Fangyuan Tian

**Affiliations:** Department of Chemistry and Biochemistry, California State University Long Beach, Long Beach, California 90840, United States

## Abstract

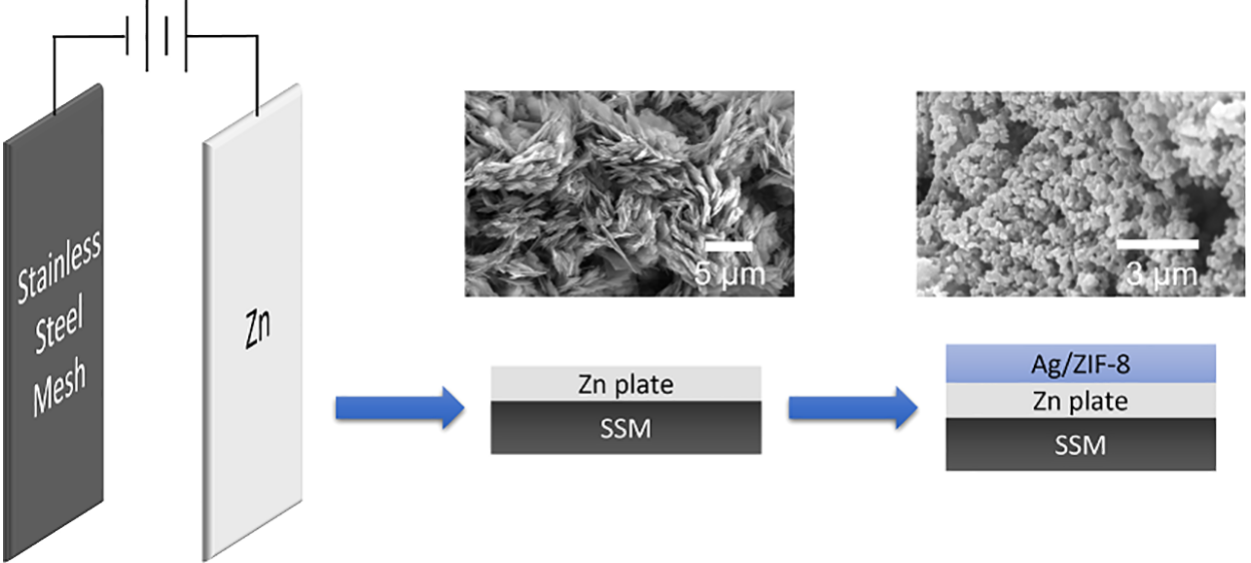

Metal–organic framework (MOF)-based membranes have been widely used in gas and liquid separation due to their porous structures and tunable compositions. Depending on the guest components, heterostructured MOFs can exhibit multiple functions. In the present work, we report a facile and rapid preparation of zeolitic imidazolate framework-8 (ZIF-8) and silver nanoparticle incorporated ZIF-8 (Ag/ZIF-8)-based membranes on stainless-steel mesh (SSM) through a “green” electrodeposition method. The SSM was first coated with a Zn-plated layer which contains mainly zinc hydroxide nitrate (Zn_5_(OH)_8_(NO_3_)_2_·2H_2_O) with a “leaf-like” morphology, providing anchoring points for the deposition of ZIF-8 and Ag/ZIF-8. It takes only 10 min to prepare a uniform coating of Zn_5_(OH)_8_(NO_3_)_2_·2H_2_O in aqueous conditions without the use of a strong base; this is by far the most efficient way of making zinc hydroxide nitrate nanocrystals. Following a similar electrodeposition approach, ZIF-8 and Ag/ZIF-8-coated SSM can be prepared within 20 min by applying a small current. The encapsulation of Ag does not alter the chemical composition nor the crystal structure of ZIF-8. The resulting ZIF-8 and Ag/ZIF-8-coated SSM have been tested for their effectiveness for rhodamine B dye removal in a fast vacuum filtration setting. Additionally, growth of *E. coli* was significantly inhibited after overnight incubation with Ag/ZIF-8-coated SSM. Overall, we demonstrate a fast synthesis procedure to make ZIF-8 and Ag/ZIF-8-coated SSM membranes for organic dye removal with excellent antimicrobial activity.

## Introduction

Degraded water quality has become a critical global issue.^1^ Water pollution exacerbates the crisis, especially in areas facing water scarcity. Common water pollutants include carcinogens, pathogens, pharmaceuticals, industrial chemicals, and microplastics.^2,3^ Wastewater is detrimental to environment and human health. Bacteria, pesticides, and nutrient-rich fertilizers can disturb already fragile ecosystems.^4,5^ The toxins in contaminated water can cause adverse health issues, such as heavy metal accumulation, hepatitis, cholera, and even cardiovascular disease.^6,7^ Therefore, efficient and effective water remediation with a relatively low cost has become an emerging priority. Typical water treatments, such as micro/nanofiltration and reverse osmosis, require excess energy and maintenance.^8^ In the last two decades, many other technologies, for example, adsorption and sediment,^9^ electro-oxidation (EO) and electro-coagulation,^10,11^ photodegradation,^12^ as well as other advanced oxidation processes (AOPs),^13^ have been exploited to remove pollutants from wastewater with relatively low operational costs while achieving excellent performance. Among all of them, the adsorption technique attracts a significant amount of attention due to its effectiveness, high stability, and reusability. Common adsorbents, such as porous carbons,^14^ zeolites,^15^ graphene,^16^ polysaccharide-based materials,^17^ ionic liquids,^18^ and metal–organic frameworks (MOFs),^19^ have been utilized for eliminating chemicals in wastewater. In particular, MOFs have exhibited great potential due to their high surface areas, versatile chemical structures, and good regeneration capabilities.

MOFs are composed of metal clusters or ions linked with organic moieties by forming three-dimensional (3D) or two-dimensional (2D) porous structures.^20−22^ MOFs and their composites are typically applied in gas capture and separation,^23,24^ catalytic reactions,^25^ fluorescence sensing,^26^ semiconductive/conductive devices,^27^ and biomedical-related fields.^28^ More recently, MOFs have been studied for wastewater treatment.^29^ Specifically, water-stable MOFs, such as pacs MOFs (CPM-243, CPM-231), MIL-100 (Al, Fe, Cr), MIL-101, UiO-66, and their derivatives, have shown great potential as adsorbents for removal of persistent organic pollutants in aqueous conditions.^30−32^ Selective pacs MOFs are ultrastable in water even in harsh acidic and alkaline conditions,^33,34^ which is critically important for treating landfill leachate. Fe-containing MILs were studied due to their potential Fenton process for degrading organics in wastewater. Besides surface adsorbing and electrostatic interactions, ZIF-8 and their composites can generate hydroxyl radicals under UV conditions to degrade organic molecules through the AOP mechanism.^35,36^ Other MOF composites, such as TiO_2_ and Ag-incorporated MOFs, have been demonstrated for photocatalytic degradation of wastewater.^37−39^ Besides bulk materials as adsorbents, MOF-modified membranes have also been explored for their potential in pollution separation in water treatment.^40,41^ However, bacteria and extracellular polymeric substances generated by microorganisms in water can cause biological fouling, which decreases the filtration performance and causes the biodegradation of membranes over time. Therefore, there is a critical need for designing MOF-based membranes with antimicrobial properties for wastewater treatment.

Selective MOFs and MOF-based materials have been demonstrated for antimicrobial applications through four mechanisms: generating antimicrobial metal ions or organic ligands; forming positively charged MOF-nanoparticles to react with bacteria or penetrating for intracellular damage; and behaving as a carrier to release antimicrobial guest agents.^42−46^ In this study, we applied two strategies to create antimicrobial silver-incorporated ZIF-8 nanoparticles (Ag/ZIF-8) on stainless-steel mesh (SSM). Based on our previous studies, ZIF-8 nanoparticles are zinc-rich with positive charge on the surface.^47^ The cell walls of both Gram-positive and Gram-negative bacteria are negatively charged.^48^ The electrostatic interactions between positively charged ZIF-8 and bacteria can disturb the cellular membrane function, leading to microorganism dysfunction. Different forms of silver, including metallic, ionic, and silver nanoparticles, have shown antibacterial activities.^49^ Basically, silver ions or silver nanoparticles (Ag NPs) can penetrate the bacteria cell membrane and bind to DNA, causing DNA damage and protein denaturation.^50^ In the presented study, we utilized Ag NPs with a diameter of 20 nm, which are still small enough to disrupt bacteria cell walls. The Ag/ZIF-8-coated SSM, in this work, was fabricated through a “green” electrodeposition method. With the development of surface coatings, including the emulsion polymerization process that Dr. B. W. Greene was involved with,^51^ membranes with high strength and predictive properties emerge. Among them, membranes that are fabricated via an electrodeposition method usually require shorter preparation time and result in a more uniform surface coverage. Here, we explored the electrodeposition of ZIF-8 and Ag/ZIF-8 on stainless-steel without polymer bindings. The resulting membranes have been tested for organic dye removal and antimicrobial properties.

## Experimental Section

### Chemicals and Materials

Stainless-steel gauze (325 mesh woven from 0.036 mm diameter wire type 316, Thermo Fisher) was cut into pieces (1 × 2 cm^2^) and used in this study. Zinc nitrate hexahydrate (Zn(NO_3_)_2_·6H_2_O, >99.0%), acetone (certified ACS grade), methanol (certified ACS grade), ethanol (200 proof), hydrogen peroxide (30%), rhodamine B (RhB, ACS reagent grade), zinc strips (2 in. × 1/4 in.), and triethylamine (99%) were purchased from Thermo Fisher Scientific. Basolite (produced by BASF) and 2-methylimidazole (>98.0%) were purchased from Sigma-Aldrich. PELCO NanoXact Silver Colloids/Nanoparticles (supplied at 1× concentration in 2 mM citrate buffer, pH 7.4) was purchased from Ted Pella. All chemicals were used as received. Milli-Q water (Millipore, resistivity = 18.2 MΩ·cm) was used for sample rinsing during the electrodeposition process.

### Cleaning of Stainless-Steel Mesh

The stainless-steel wire gauze was pretreated via ultrasonication for 10 min in a solution with a ratio of 1:1:1 of 200 proof ethanol, acetone, and hydrogen peroxide at 75 °C. Upon completion, the stainless-steel gauze was thoroughly washed with DI water three times, followed by drying with nitrogen gas. Then, it was placed in a UV-Ozone cleaner (Bioforce Nanosciences) for 10 min on each side before storage in a clean glass Petri dish in a desiccator.

### Zinc Deposition on Stainless-Steel Mesh

The electrochemical cell for Zn plating was prepared as follows: a Zn strip was applied as the anode (connecting to the positive terminal) and the pretreated stainless-steel mesh (SSM) as the cathode (connecting to the negative terminal) in a 1 M Zn (NO_3_)_2_·6H_2_O aqueous solution as electrolyte. A current (0.01 A per cm^2^) was applied on the electrochemical cell, and 10 min allowed for the reaction to complete. Once the reaction was terminated, the SSM was washed with Milli-Q water and dried with nitrogen; it was then further dried in a desiccator for 24 h.

### Electrodeposition of Ag/ZIF-8 on Modified Stainless-Steel Mesh

First, colloidally capped silver nanoparticle (Ag NP)-doped ZIF-8 electrolyte was prepared by mixing Zn (NO_3_)_2_·6H_2_O, triethylamine, 2-methylimidazole, and Ag NPs with an average particle size of 20 nm. More specifically, 1.476 g of Zn(NO_3_)_2_·6H_2_O was dissolved in 100 mL of methanol with 5 mL of Ag NP solution; it was then mixed with 3.266 g of 2-methylimidazole in 100 mL of methanol with 6 mL of triethylamine. The resulting solution was then stirred for 1 h and was washed three times with methanol to remove excess triethylamine and unreacted Ag nanoparticles. The resulting solution was then used as the electrolyte in the electrodeposition cell. Second, the electrodeposition cell contains the Zn-plated SSM placed as the cathode, while a Zn strip was placed as the anode. A current of 0.01 A per cm^2^ was applied across both electrodes for 20 min. Once the reaction was completed, the Ag/ZIF-8-coated SSM sample was washed with ethanol and dried with nitrogen; it was then left to further dry in a desiccator for 24 h.

### Characterization

#### Infrared (IR) Spectroscopy

An attenuated total reflectance infrared (ATR-IR) spectroscopy (Bruker Instruments, Alpha I Platinum) was used to measure the transmittance of the mesh samples to confirm their chemical compositions. All IR spectra were collected in the range of 4000–400 cm^–1^ at a resolution of 8 cm^–1^ with a total of 128 scans per spectrum with open air as the background.

#### Powder X-ray Diffraction (XRD)

Powder X-ray analysis was performed on a Bruker D2 Phaser diffractometer (G2) with a Cu Kα radiation source. The acquisitions were carried out in the 2θ range of 5°–70° with a step size of 0.02°. Each testing SSM sample was fixed on a piece of microscopic glass slide (1.5 × 1.5 cm) using a PELCO tab (Ted Pella, Inc.) before loading to the PMMA ring holder for XRD analysis.

#### Scanning Electron Microscopy (SEM)

SEM images were obtained using a Phenom ProX G6 instrument equipped with energy dispersive X-ray spectroscopy (EDS) analysis. The images were taken with an acceleration voltage of 15 kV and a working distance between 5 and 10 mm in vacuum conditions. Prior to imaging, ZIF-8 and Ag/ZIF-8-coated SSM samples were sputter-coated with a thin layer of gold and palladium for better resolution. Samples used for EDS analysis were not sputter-coated to preserve all elemental information.

#### UV–Vis Spectroscopy

A spectrophotometer (VWR model no. UV-1600PC) was used to measure the optical density of the bacteria culture solution before and after the antimicrobial testing on our samples, including bare clean SSM, Zn-plated SSM, ZIF-8-coated SSM, and Ag/ZIF-8-coated SSM. The absorbance of RhB was also measured using the same spectrophotometer before and after being filtered by our mesh samples.

### Rhodamine B Filtration Testing

A 20 ppm aqueous solution of RhB was prepared as stock solution, and it was then diluted to 1 ppm with DI water. Filtration of the RhB solution was achieved through the following scheme: A circular 1 cm diameter of each SSM sample, including bare clean SSM, Zn-plated SSM, ZIF-8 and Ag/ZIF-8-coated SSM, was held in place by a cap with an opening, as shown in the Supporting Information. A 10 mL sample of the 1 ppm RhB solution was applied with a clean syringe through the filter and was then collected into the flask that was connected to an in-house vacuum line. The absorbance of filtered RhB solution was analyzed via UV–vis spectroscopy. Each data point was calculated from three replicates.

### Bacterial Growth Measurement

Competent *Escherichia coli* (*E. coli*) cells (New England Biolabs, catalog no. C2987H) were transformed with pcDNA3.1 plasmid vector encoding an ampicillin resistance gene. The glycerol stock was used to inoculate Luria–Bertani (LB) broth supplemented with 100 ug/mL ampicillin (Amp), and the culture was grown for 16 h in a shaking incubator (225 rpm) at 37 °C. Next day, the overnight culture was diluted to A_600_ of 0.1/mL in 200 mL of fresh LB-Amp medium and grown for 1 h in a shaking incubator (225 rpm) at 37 °C. The culture was then distributed evenly in sterile flasks containing either the bare SSM, Zn-plated SSM, ZIF-8-coated SSM, or Ag/ZIF-8-coated SSM. These cultures were allowed to grow overnight under the same growth conditions, and the A_600_ was measured to determine bacterial growth in each flask. All experiments were repeated three times.

## Results and Discussion

### Design and Characterization of Ag/ZIF-8-Coated SSM

**Scheme 1:**
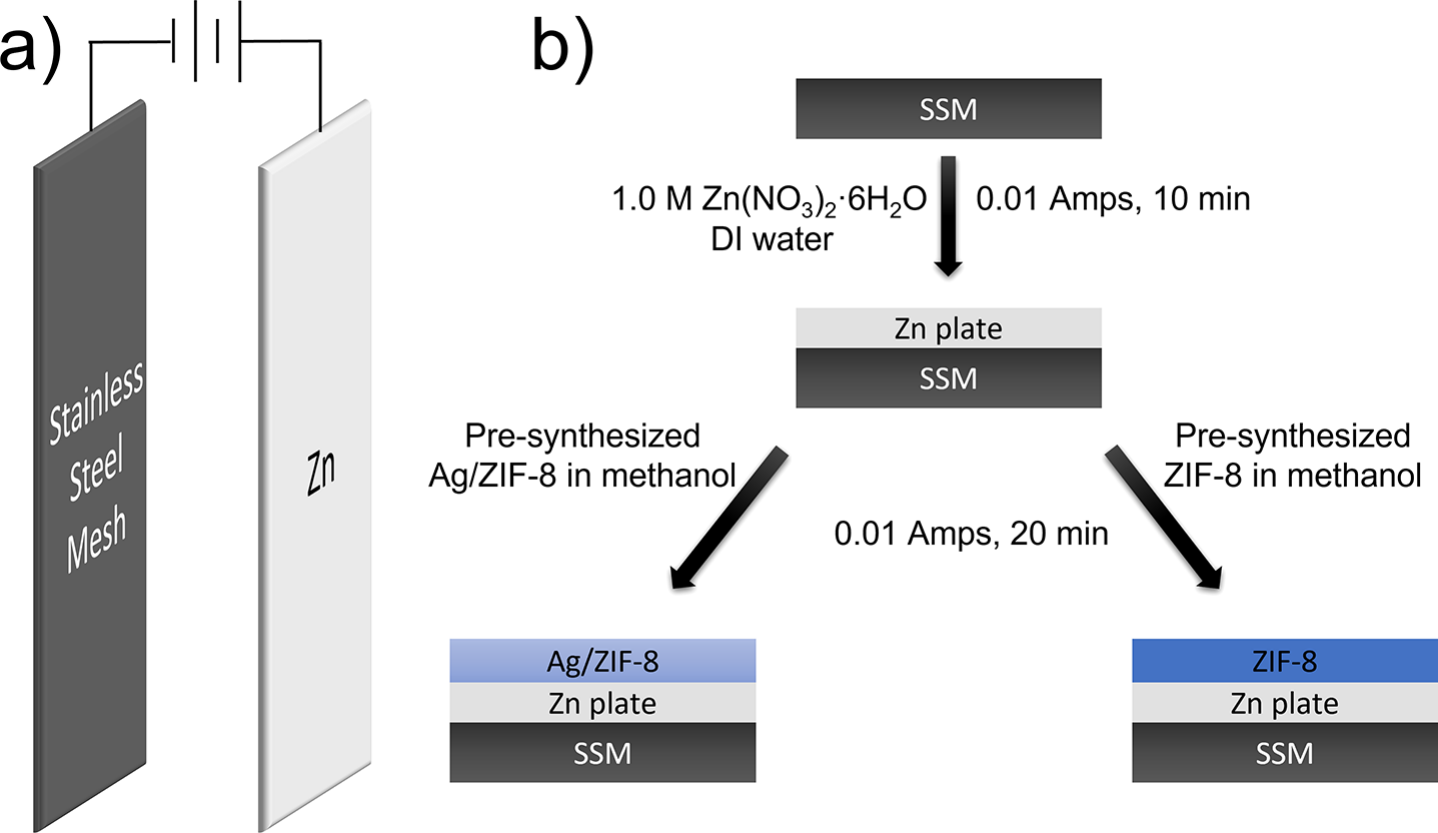
(a) Schematic Illustration of the Electrodeposition Process: Bare or Functionalized Stainless-Steel Mesh (SSM) as Cathode and Zinc Strip as Anode; (b) Schematic Representation of the Preparation Steps to Prepare ZIF-8 and Ag/ZIF-8-Coated SSM Membranes The size of the electrodes and the thickness of each layer are not drawn to scale.

For reliable applications of MOF-based antimicrobial water remediation, stability in aqueous environment is critical. ZIF-8 has been known for its hydrophobicity and thermal and chemical stability in various conditions,^52^ which makes it a great candidate as adsorbent for water treatment. The porous structure of ZIF-8 exposes excess cavities for loading guest molecules for catalytic conversion in aqueous conditions.^53,54^ Additionally, ZIF-8 has multiple surface groups, including hydroxide, carbonates, and amines, which promote surface adsorption for organics.^47^ Moreover, hydroxyl radicals can be generated by ZIF-8 under UV conditions, enabling fast reactive oxidizing of organics in water treatment.^35^ However, bulk ZIF-8 has its limitation as recollection and regeneration of the powder format is difficult, and there is also a high maintenance cost for the facility for dealing with slurry adsorbents.^2^ Therefore, fixing ZIF-8 on a substrate is practically important. To design surface-supported ZIF-8 filtration systems, we have considered the following aspects: (A) stability in aqueous conditions; (B) performance with multiple functions; and (C) mass scale production. After comparing different substrates, including cotton, fiber, glass frit, α-alumina, and polymer membrane, we selected 316 type SSM for growing ZIF-8 and its composite mainly due to its inertness in aqueous environments while maintaining good conductivity for electrodeposition. The complete membrane fabrication process is illustrated in Scheme 1.

**Figure 1:**
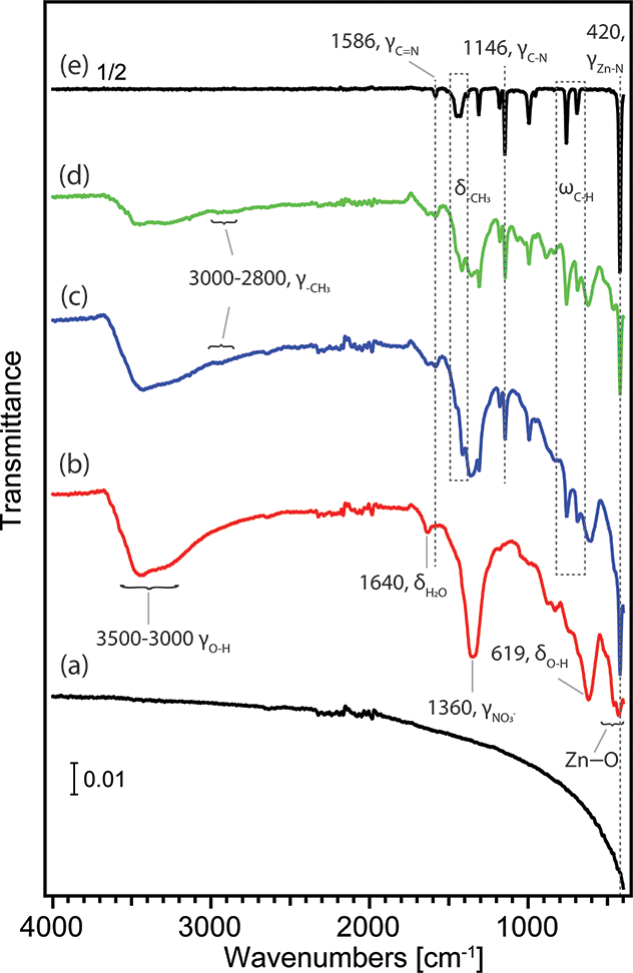
ATR-IR spectra of (a) clean stainless-steel mesh (SSM), (b) Zn-plated SSM, (c) ZIF-8-coated functionalized SSM, (d) Ag/ZIF-8-coated functionalized SSM, and (e) basolite powder. All spectra used air as background. The transmittance intensity of the spectrum of basolite was divided by a factor of 1/2 for comparison in the same scale with other spectra.

**Figure 2:**
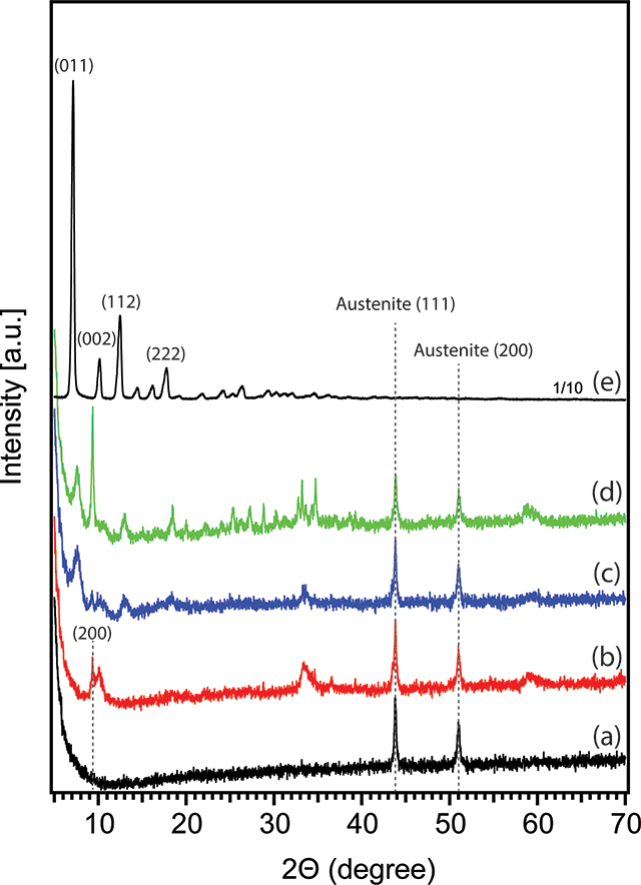
X-ray diffraction (XRD) patterns of (a) clean stainless-steel mesh (SSM), (b) Zn-plated SSM, (c) ZIF-8-coated functionalized SSM, (d) Ag/ZIF-8-coated functionalized SSM, and (e) basolite powder. The intensity of the basolite result was divided by a factor of 1/10 for comparison in the same scale with others.

The SSM used in this study was treated with both wet-chemistry and dry-cleaning methods to remove any organic contaminants on both sides. After the cleaning process, the SSM shows no obvious carbonates or other organic features based on the IR analysis, as shown in Figure 1a. The XRD pattern of clean SSM is shown in Figure 2a; two peaks were observed at 43.8°and 51.0°, which are attributed to the (111) and (200) phase of austenite (γ-Fe, an allotrope of iron), respectively.^55^ Based on our preliminary studies, direct electrodeposition of ZIF-8 on SSM resulted in poor adhesion; thus, the adsorbed ZIF-8 nanocrystals were easily scratched off. To increase the durability, we first galvanized the SSM with a zinc strip by applying a current of 0.01 A per cm^2^ in a 1 M Zn (NO_3_)_2_·6H_2_O aqueous solution at room temperature. This Zn-plating method results in robust ZIF-8 and Ag/ZIF-8 layers on the SSM surfaces. To determine the chemical composition of the Zn-layer on SSM, we compared the IR spectrum collected on the sample (Figure 1b) with several references, including the spectra of zinc oxide (ZnO, Figure S1), zinc hydroxide (ε-Zn(OH)_2_),^56^ zinc nitrate (Zn(NO_3_)_2_),^57^ and zinc hydroxide nitrate (Zn_5_(OH)_8_(NO_3_)_2_).^58^ As presented in Figure 1b, a broad peak was observed around 3450 cm^–1^, attributed to the stretching vibration of hydroxide.^59^ A small peak at 1640 cm^–1^ is associated with the bending vibration of water molecules^60^ that may be intercalated in the Zn layer during the electrodeposition process. We noticed an intensive peak at 1360 cm^–1^ which was assigned to the υ_3_ vibrational mode of NO_3_^–^ with *D*_3*h*_ symmetry.^56,59,61^ A band at 619 cm^–1^ and a weak peak at 522 cm^–1^ are due to the bending vibrations of hydroxide.^56^ In addition, the features shown at 463 and 432 cm^–1^ are related to the lattice vibrations of Zn–O bonds.^62^ Based on our observations, the Zn layer coated on SSM obtained through electrodeposition could be zinc hydroxide nitrate hydrate or a mixture of zinc hydroxide and nitrate hydrates. The IR study alone cannot exclusively confirm the chemical composition of the Zn-layer. Therefore, we turned to X-ray diffraction studies to check its crystal structure.

**Figure 3:**
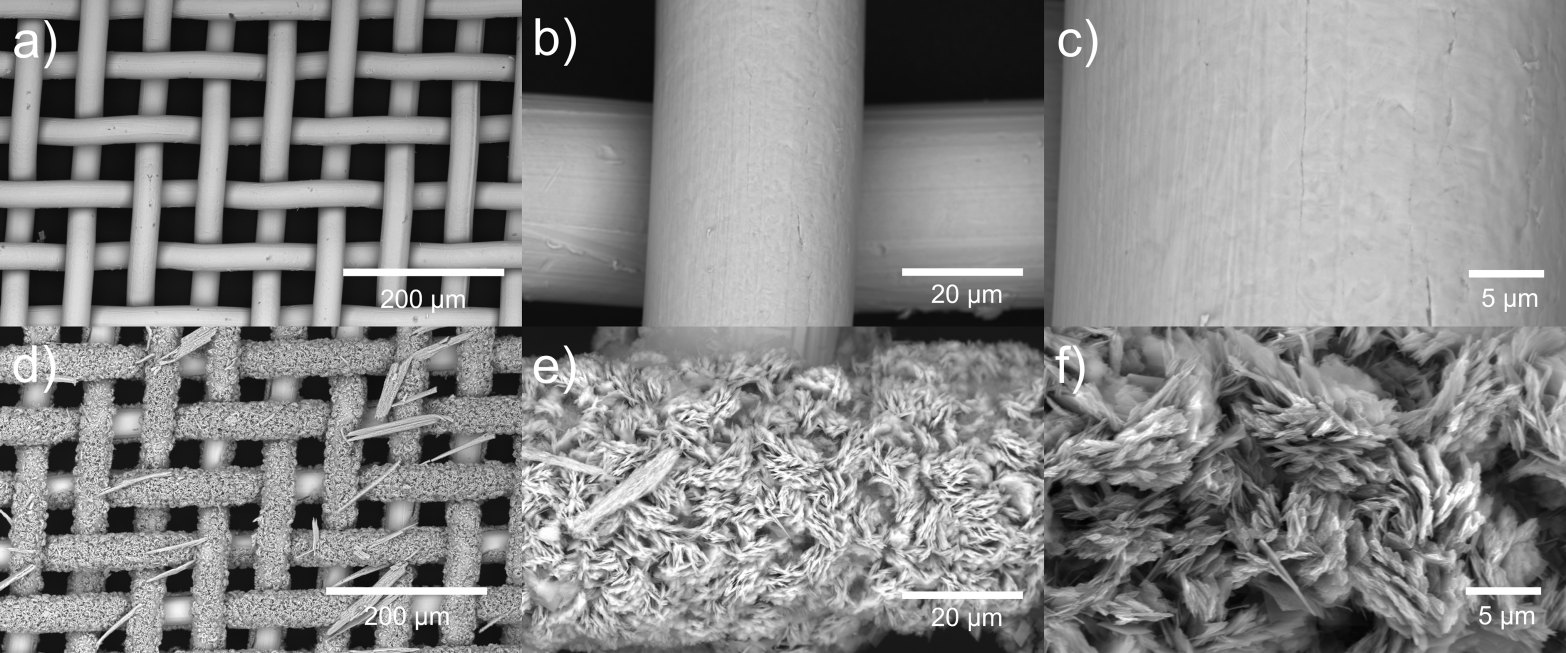
SEM images of clean stainless-steel mesh (SSM, a–c) and Zn-plated SSM (d–f).

Figure 2b presents the XRD pattern of the SSM after Zn-plating. Besides the peaks at 41.8°and 51.0° from the stainless-steel substrate, the peak at 9.3° is the characteristic (200) phase of the monoclinic structure of Zn_5_(OH)_8_(NO_3_)_2_·2H_2_O.^63,64^ Another peak observed at 10.1° is due to the layered double hydroxide with a basal spacing of interlayer nitrate anions from the electrolyte solution.^59,61^ Two broad reflections around 33.5° and 59.1° can be related to ε-Zn(OH)_2_ and/or a mixture of layered double hydroxide with Zn_5_(OH)_8_(NO_3_)_2_·2H_2_O.^56,58^ Last, we studied the morphology of SSM after the Zn-plating. Figure 3 shows the SEM images of SSM before and after the Zn plating electro-treatment. The surface of bare SSM is smooth and flat, as shown in Figure 3a–c. After Zn-plating, we noticed a few long fibers on the surface (Figures 3d and S3d), which could be related to a zinc species that was degraded from the Zn electrode during electrodeposition. The majority of the SSM surface was covered with sheet-like crystals (Figure 3f), which is known to be the morphology of Zn_5_(OH)_8_(NO_3_)_2_ crystals.^58^ We also compared it with the ε-Zn(OH)_2_ crystals, usually demonstrating a truncated octahedral shape,^65,66^ which was not observed on our samples. Therefore, by combining IR, XRD, and SEM studies, we confirmed that the Zn-plated SSM was mainly covered with Zn_5_(OH)_8_(NO_3_)_2_·2H_2_O with some water molecules and nitrate ions intercalated in between the crystal sheets. To our knowledge, this is by far the most convenient and the fastest synthesis approach to produce Zn_5_(OH)_8_(NO_3_)_2_·2H_2_O crystals; no harsh chemicals or heating is needed in our electrodeposition method. Studying the underlying Zn layer is critically important to understand how ZIF-8 and its composite can be attached to the functionalized surfaces.

After confirming a uniform coating of Zn_5_(OH)_8_(NO_3_)_2_·2H_2_O was prepared, we used the functionalized SSM as cathode and immersed it in a presynthesized Ag/ZIF-8 methanol solution as electrolyte by applying a current for depositing Ag/ZIF-8 nanocrystals on the SSM. As a comparison, we also applied the same strategy for attaching ZIF-8 nanocrystals on functionalized SSM by using a premade ZIF-8 methanol solution as electrolyte. In both cases, a Zn strip was used as the anode to provide free Zn^2+^ ions. The presynthesized ZIF-8 solution was prepared by following previously reported procedures^35,67,68^ with some modifications. Triethylamine was added to the 2-methylimidazole solution to fully deprotonate the imidazole ligands, thus promoting ZIF-8 formation in a short period of time. Ag NPs were introduced into the Zn (NO_3_)_2_·6H_2_O methanol solution before zinc salt and imidazole had been mixed. Panels c and d of Figure 1 present the IR spectra of SSM after being coated with ZIF-8 and Ag/ZIF-8, respectively. As a comparison, we recorded an IR spectrum of Basolite, commercially available ZIF-8 manufactured by BASF, as shown in Figure 1e. The observed IR frequencies of the ZIF-8 and Ag/ZIF-8-coated SSM are found to be consistent with the ones taken from commercial ZIF-8 and previously reported in the literature.^69,70^ The peak at 420 cm^–1^ is contributed from the vibrational stretching of Zn–N formed between tetrahedrally coordinated zinc ions and 2-methylimidazole ligands.^71,72^ In the fingerprint region, multiple features at 693, 759, and 1311 cm^–1^ correspond to the C–H bending and rocking in the imidazole ring, and the peaks between 900 and 1000 cm^–1^ are related to the rocking vibrations of −CH_3_ on 2-methylimidazole.^73^ The C–H bending of −CH_3_ and C–C stretching was noticed on a doublet around 1449 cm^–1^.^73^ An intensive peak at 1146 was attributed to the C–N stretching in the imidazole ring, which is a characteristic feature observed in ZIF-8.^71,74^ Another small band at 1586 cm^–1^ is associated with the stretching of C=N also from the imidazole ring.^71,74^ The peaks at 1312, 1183, and 998 cm^–1^ are all related to the symmetric/asymmetric and out-of-plane bending of C=C–N from the imidazole ring.^73^ Based on our IR studies, we confirmed that both ZIF-8 and Ag/ZIF-8 were successfully attached to the prefunctionalized SSM. More importantly, Ag doping did not change the chemical composition of ZIF-8.

Next, we examined the crystal structures of the formed ZIF-8 and Ag/ZIF-8 layers and compared them with the XRD pattern taken from commercial ZIF-8 (Figure 2e) and the reported literature.^47,67^ Panels c and d of Figure 2 exhibit the XRD patterns of the SSM after being modified with ZIF-8 and Ag/ZIF-8, respectively. We noticed the signature peak around 7.5°, which is due to the (011) phase of ZIF-8.^71^ The 2θ at 9.4°, 12.9°, and 18.4° correspond to the orientations of (002), (112), and (222) in ZIF-8.^67,71^ Although the peaks were noticed to shift by 0.8° compared with the pattern obtained on Basolite, we believe this is due to the height of SSM samples during measurements. Unlike powder samples, SSM samples were secured on a glass slide before the X-ray analysis; the height of our meshes was slightly higher than the powder sample. Overall, our XRD studies also confirmed the ZIF-8 layer was successfully attached to the Zn-plated SSM through electrodeposition, and ZIF-8 retains its crystallinity with Ag incorporation.

**Figure 4:**
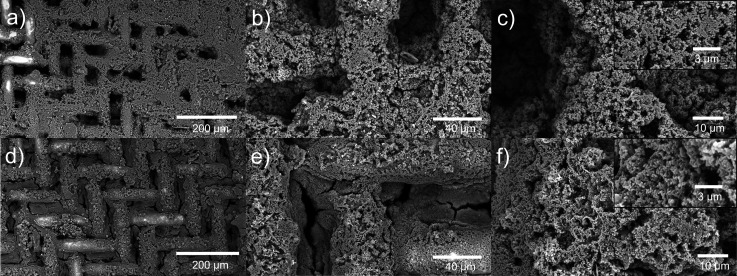
SEM images of stainless-steel mesh (SSM) coated with ZIF-8 (a–c) and with Ag/ZIF-8 (d–f). Inset images are the zoomed-in regions.

The morphology of the SSM after each modification step was monitored by SEM. As shown in Figure 4, after a coating of ZIF-8 and Ag/ZIF-8, the surfaces of SSM were covered with a dense layer of crystals with spherical shapes, which aligns well with previously reported ZIF-8 nanocrystals.^47,75,76^ More importantly, the Ag/ZIF-8 layer exhibited a topology similar to that of the ZIF-8 coating, indicating the Ag doping did not alter the ZIF-8 morphology. ZIF-8 has a Zn-rich surface with positive charge that will be attracted to the cathode during the electrodeposition process. Furthermore, the “sheet-like” zinc hydroxide nitrated covered Zn-plated SSM can provide a higher surface area for depositing ZIF-8 and Ag/ZIF-8 particles.

**Figure 5:**
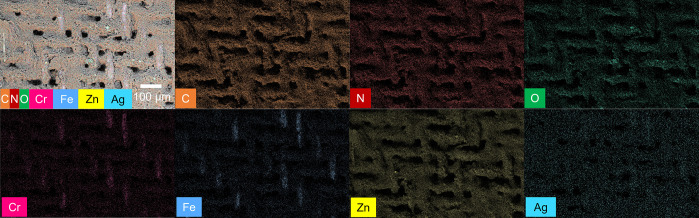
SEM image and EDS mapping of the same region on Ag/ZIF-8-coated SSM. The elemental mapping of C (orange), N (red), O (green), Cr (magenta), Fe (blue), Zn (yellow), and Ag (teal). The SEM image shows all elements overlaid.

In addition, we performed EDS analysis to study the elemental information on the SSM after each coating process. Specifically, we aim to deteremine the presence of Ag, and possibly its location, in ZIF-8. The EDS results of clean, Zn-plated, and ZIF-8-coated SSM are summarized in the Supporting Information. For bare clean SSM, several elements were detected, including Fe (61.4 wt %), Cr (16.8 wt %), Ni (11.9 wt %), C (8.2 wt %), Mo (1.6 wt %), and a trace amount of Mn, which is consistent with the manufacturer’s information. Based on our IR, XRD, and SEM studies, we have confirmed that the Zn-plated SSM was mainly covered by zinc hydroxide nitrate, which was also supported by our EDS results: we found significant amounts of Zn and O along with N, and small amounts of Fe, Ni, and C from the SSM substrate, on the Zn-plated SSM. After the ZIF-8 coating, the composition of C and N has increased to 25.2 and 45.4 wt %, respectively, due to the 2-methylimidazole ligands in ZIF-8. The atomic concentration between Zn and N is approximately 1:12, which is greater than the 1:4 stoichiometry in ZIF-8. We think that is due to the sublayer of zinc hydroxide nitrate crystals. Figure 5 presents the EDS elemental mapping of a large area of Ag/ZIF-8-coated SSM. The elemental composition and their percentage are listed in Table S1. We noticed that Ag was distributed across the whole SSM, overlaying well with the ZIF-8 crystals. Based on the atomic concentrations of Ag and Zn, we calculated about 3.4% of Ag was doped with ZIF-8. Considering the aperture diameter of ZIF-8 is around 3.4 Å,^77^ which is much smaller than the size of Ag NPs (∼20 nm), we think Ag NPs cannot enter the cages of ZIF-8. Instead, they could be mixed inside of ZIF-8 particles and/or be adsorbed on the surface of ZIF-8. A similar encapsulation of Ag inside of ZIF-8 nanoparticles were reported by Jiang et al.^78^ Since the electron beam penetration depth is about 10 μm in EDS analysis, we were unable to distinguish between surface adsorbed and shelled Ag species by this technique alone. Once we confirmed the ZIF-8 and its composite has been attached to the functionalized SSM, we decided to study their performance in water remediation and the antimicrobial properties.

### ZIF-8 and Ag/ZIF-8 Membranes for Rhodamine B Removal

**Figure 6:**
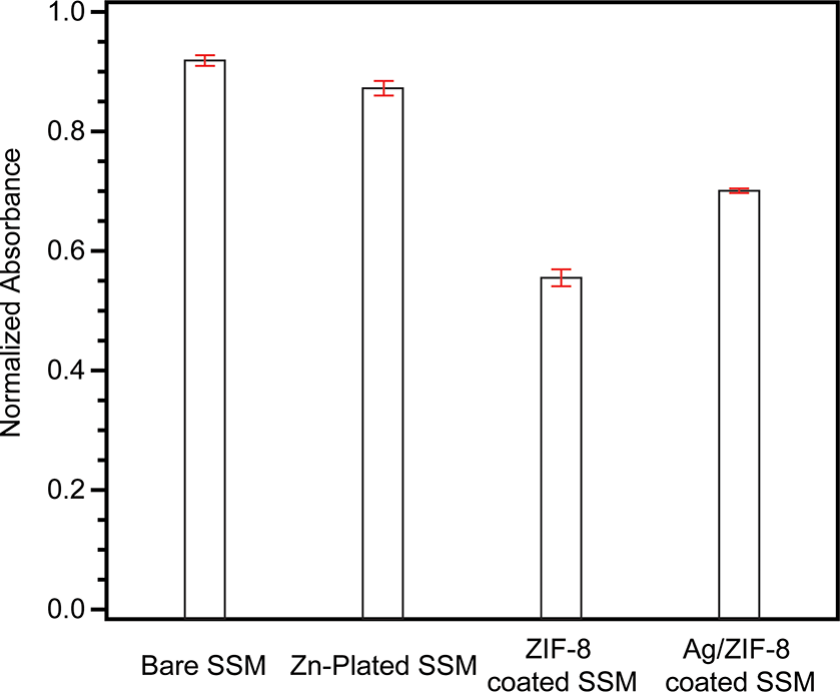
Normalized UV–vis absorbance of rhodamine B (RhB) in water after being filtered by bare, Zn-plated, ZIF-8-coated, and Ag/ZIF-8-coated SSM. Error bars indicate standard deviation, *N* = 3.

Adsorption and degradation of RhB by ZIF-8 has been studied by several groups.^35,79−82^ The goal of this part is to examine whether our ZIF-8 and Ag/ZIF-8 membranes can remove organic dye (RhB as a model) to the same extent as its bulk format. We are also interested in evaluating the filtration limit of our prepared membranes. Typically, it is difficult to remove pollutants when their concentrations are low. Therefore, we used an extremely low concentration of RhB (1 ppm) for testing the filtration effectiveness. The absorbance of RhB was measured before and after filtration at 561 nm by a UV–vis spectrometer. The filtration testing was carried out on all four types of samples: bare clean SSM, Zn-plated SSM, and ZIF-8- and Ag/ZIF-8-coated SSM. All absorbance readings were normalized to the value of the RhB solution before filtration, as shown in Figure 6. For bare SSM, the absorbance of the filtered solution decreased by about 8%. Zn-plated SSM shows a slightly lower absorbance of RhB compared to the bare SSM. We noticed that the normalized absorbance of the filtered RhB solution by ZIF-8-coated SSM was 0.55 ± 0.01, indicating almost half of the RhB was removed after a single filtration. Ag/ZIF-8-coated SSM also exhibited a 30% drop in absorbance of RhB, suggesting some of RhB molecules can be trapped by the surface supportive Ag/ZIF-8 membrane. In our previous study, powder ZIF-8 nanocrystals were shown to adsorb about 40% of RhB after 1 min stirring in the dark.^35^ Our ZIF-8 and Ag/ZIF-8 membranes exhibited a similar removal rate, but the filtration process is much faster with the aid of vacuum in our system. The removal rate of RhB by our ZIF-8-based membrane is in line with other previously reported membrane materials, for example, GO-PDA/PES-SPES (40% at 5 ppm),^83^ coal-based carbon membrane (70% after 3 h of treatment),^84^ and CCA/Pd-TiO_2_ polysulfone membranes (70.8–80.4%),^85^ but the eluent permeation rate is much faster in our case.

### Antimicrobial Property Evaluations

**Figure 7:**
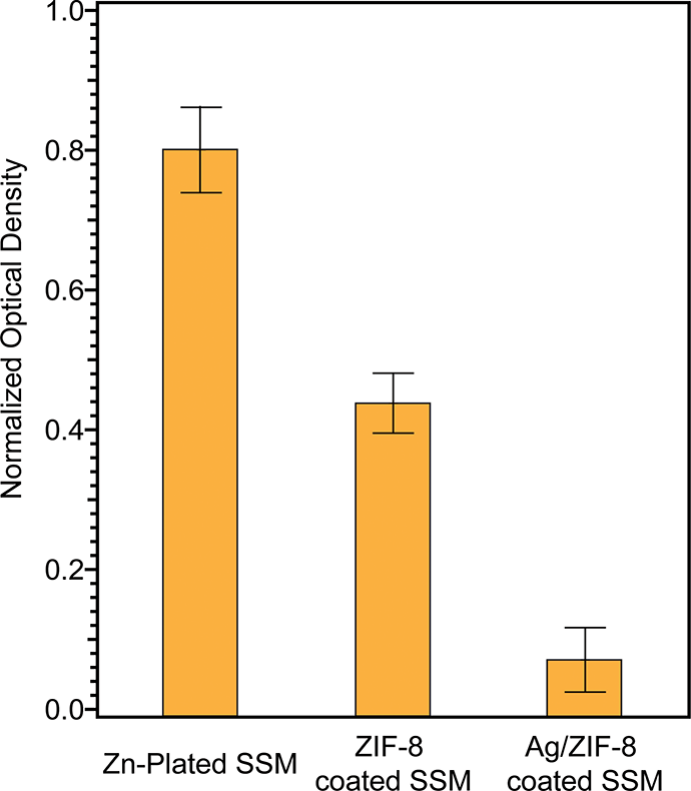
Growth comparisons among bacterial cultures grown in the presence of Zn-plated, ZIF-8-coated, and Ag/ZIF-8-coated SSM. All readings were normalized to the optical density measured on the control culture incubated with bare clean SSM. Error bars indicate standard deviation, *N* = 3.

One of the challenges of using membranes for water treatment is bacterial biofilm formation.^40^ To evaluate the antimicrobial properties of ZIF-8 and Ag/ZIF-8-coated SSM, we tested their effect on bacterial growth. Figure 7 shows the comparison of optical density (OD) of the *E. coli* cultures measured at 600 nm after overnight incubation with the indicated SSM. The OD readings for cultures containing Zn-plated, ZIF-8-coated, and Ag/ZIF-8-coated SSM were normalized to the OD obtained from *E. coli* culture incubated with bare clean SSM. As shown in Figure 7, the Zn-plated SSM shows weak antimicrobial behavior with 80 ± 6% growth compared to the control SSM. The growth was reduced to 43 ± 4% after culturing with ZIF-8-coated SSM under the same conditions. Previous studies have reported the antimicrobial properties of ZIF-8 mainly due to the free Zn^2+^ ions on the surface that can kill bacteria.^39,42^ Lastly, only ∼7% growth was observed after incubation with Ag/ZIF-8-coated SSM. These results indicate an effective antimicrobial property of Ag/ZIF-8-coated SSM membrane. This can be due to the Ag NP-mediated damage to the DNA and/or proteins. A similar antimicrobial activity was also reported on Ag@ZIF-8 heterostructure nanowires.^46^ This result also suggests that at least some Ag NPs were adsorbed on the surface of ZIF-8 particles.

## Conclusions

In the present work, we report an electrodeposition approach to prepare ZIF-8 and Ag/ZIF-8-coated SSM membranes that can be used for water remediation and have antimicrobial activity. To foster the stability and durability of the ZIF-8 and Ag/ZIF-8 coatings, we first fabricated a Zn-plated coating using electrodeposition; the resulting layer was confirmed to be zinc hydroxide nitrate nanocrystals, which provides high surface area for adsorbing ZIF-8 and Ag/ZIF-8 particles afterward. The prepared ZIF-8 and Ag/ZIF-8 modified SSM enable the removal of RhB during a fast vacuum filtration process. In addition, both ZIF-8 and Ag/ZIF-8-coated SSM samples exhibit antimicrobial properties with *E. coli* as a model bacterium, demonstrating great potential for water treatment. Further membrane manufacturing and detection limit measurements can be adopted to improve their performance in future studies. In addition, the Ag/ZIF-8 heterostructures can be further investigated in the field of optical plasmonic materials.

## Supporting Information Available

Additional IR, EDS, SEM, and UV–vis studies (PDF)
