# Supplementary material for: Electrodeposition of Ag/ZIF-8-Modified Membrane for Water Remediation
Source: Langmuir. 2023 Jan 30;39(6):2291–300. doi: 10.1021/acs.langmuir.2c02947 (PMC9933538; doi:10.1021/acs.langmuir.2c02947)
Supplement: Supplementary file 1 — la2c02947_si_001.pdf [file la2c02947_si_001.pdf]

Electrodeposition of Ag/ZIF-8 modified membrane for water remediation

*Ricky Rodriguez, Miguel S. Palma, Deepali Bhandari, Fangyuan Tian**

Department of Chemistry and Biochemistry, California State University Long Beach, Long Beach, CA, USA 90840

*Corresponding author: fangyuan.tian@csulb.edu

Supporting Information

Table of Contents

1. Material characterization…………………………….……..………………………*S-2*

2. Water remediation testing..…..……………………………………..…………………*S-8*

1. Material characterization.

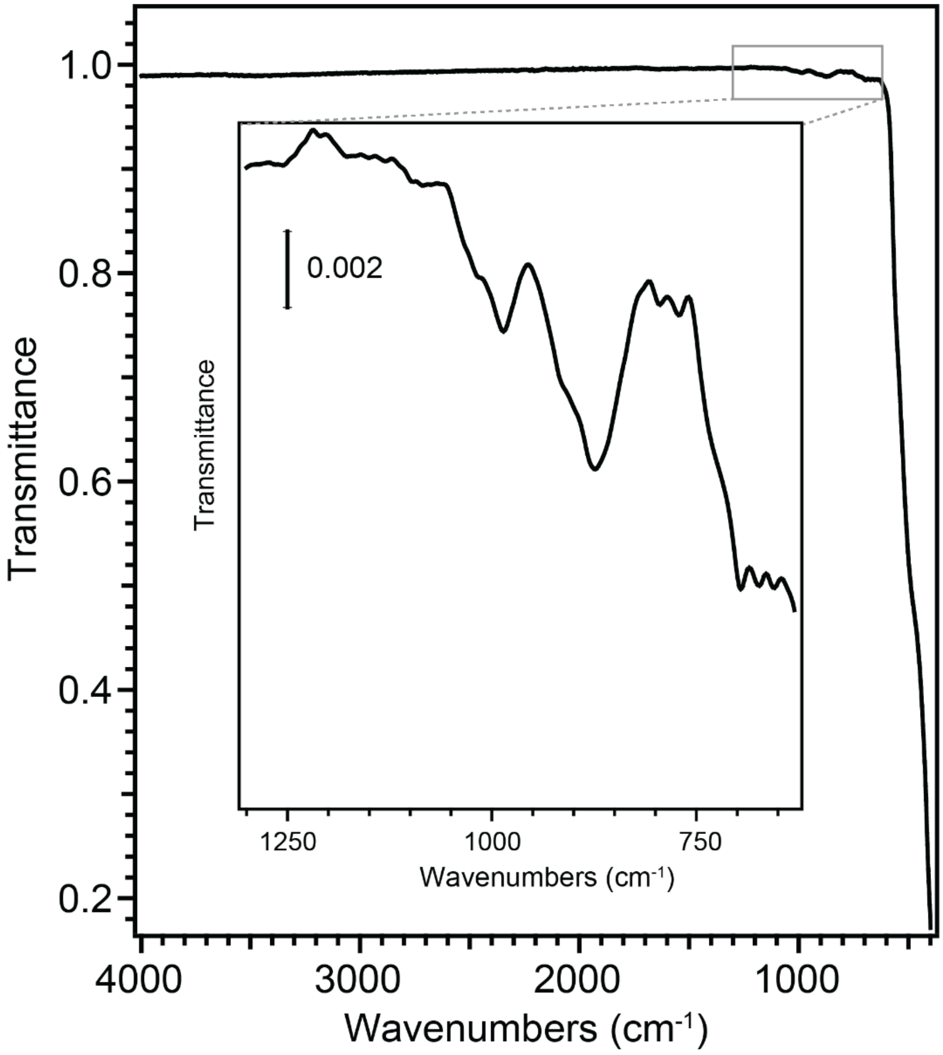

Figure S1. ATR-IR spectrum of ZnO powder in the range of 4000 to 400 cm^{-1}, the inset is the zoom-in region between 1300 and 630 cm^{-1}. The Zn-O stretch was observed around 400 cm^{-1}.

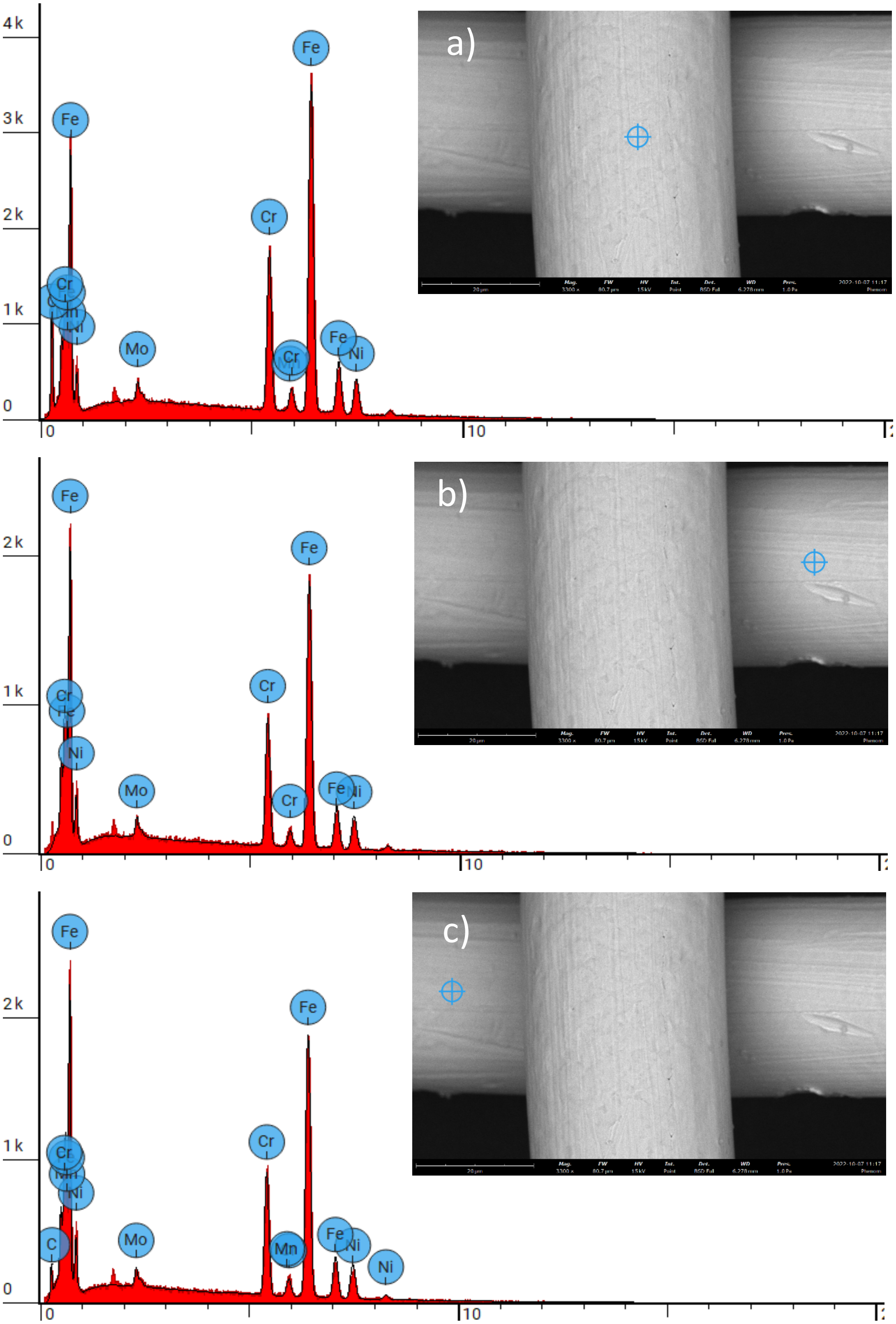

Figure S2. SEM images and EDS spectra of three spots (a-c) on a clean SSM.

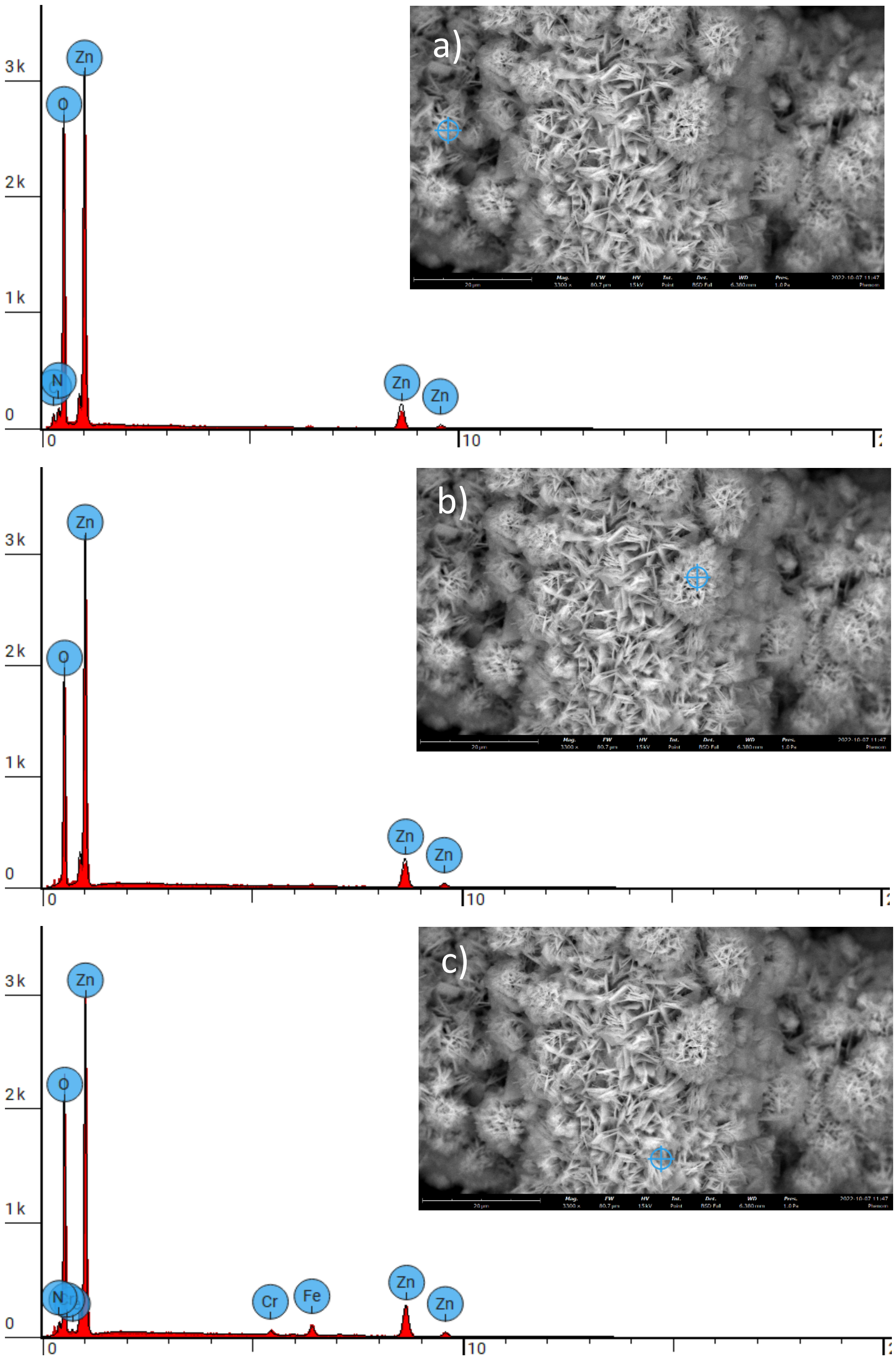
a)
b)
c)
3 k
2 k
1 k
0
10
Zn
O
N
Cr
Fe

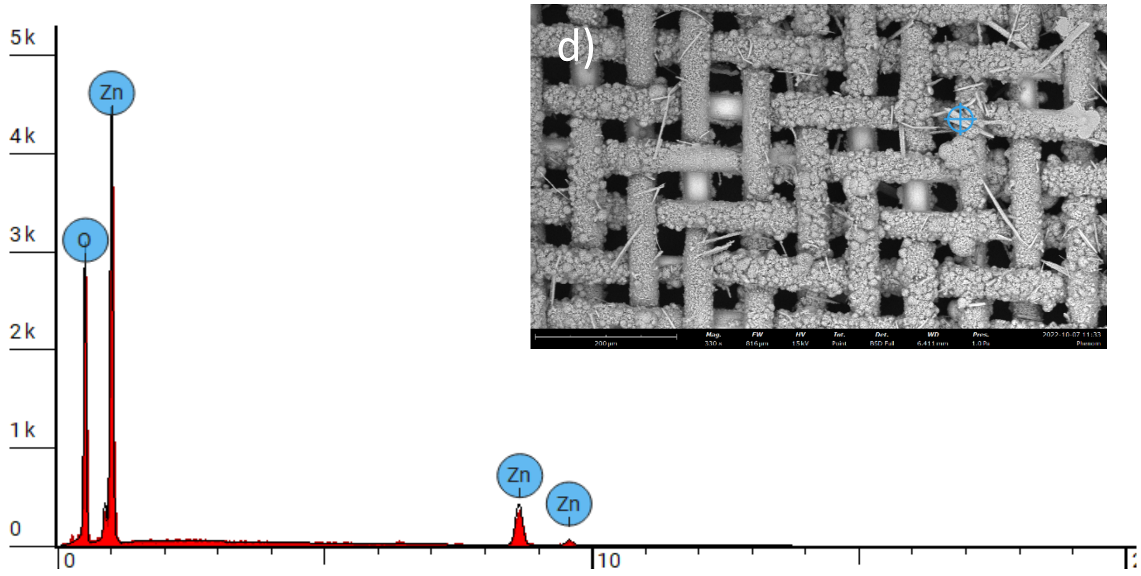

Figure S3. SEM images and EDS spectra of four spots (a-d) on Zn-plated SSM.

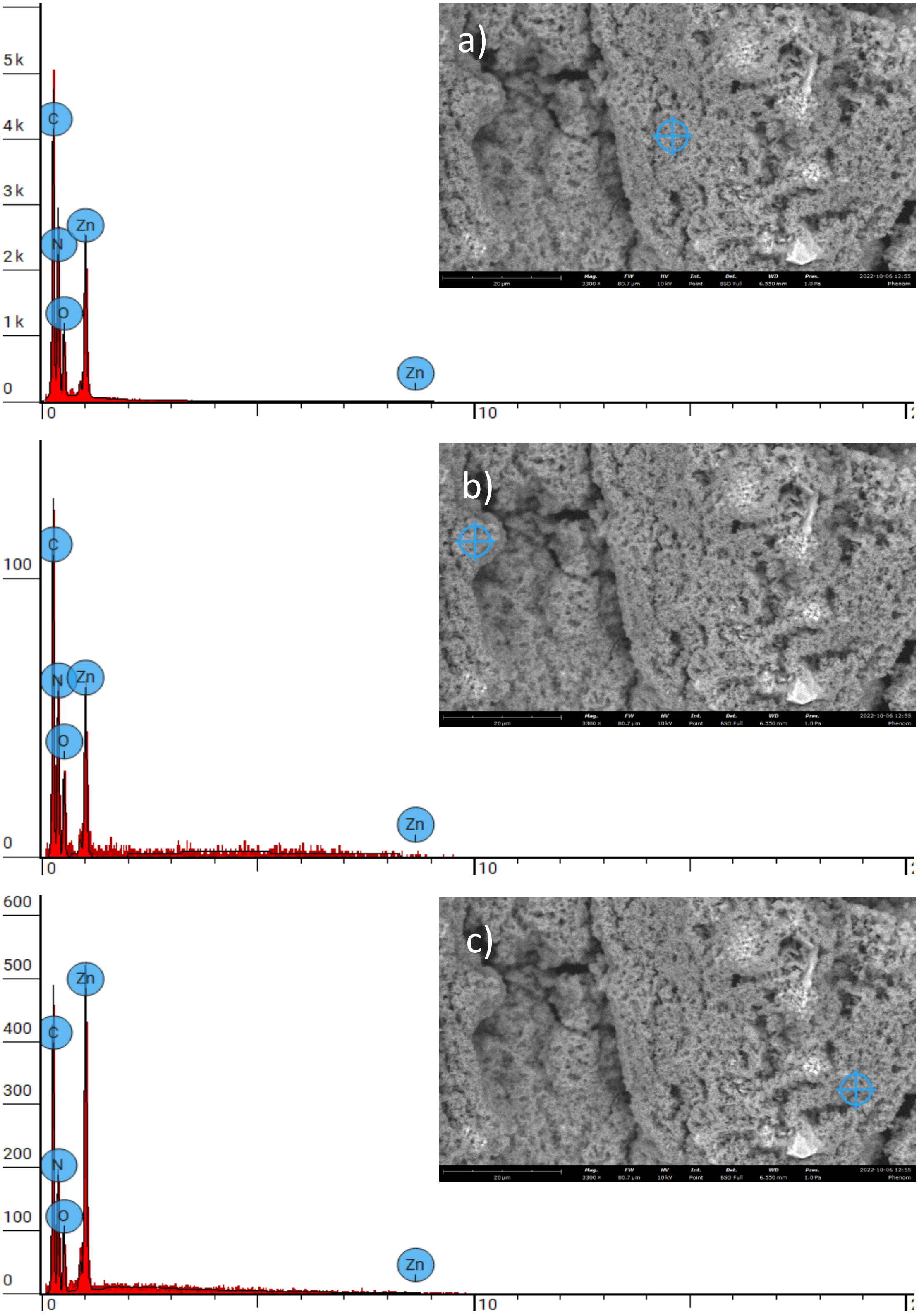

Figure S4. SEM images and EDS spectra of three spots (a-c) on ZIF-8 coated SSM.

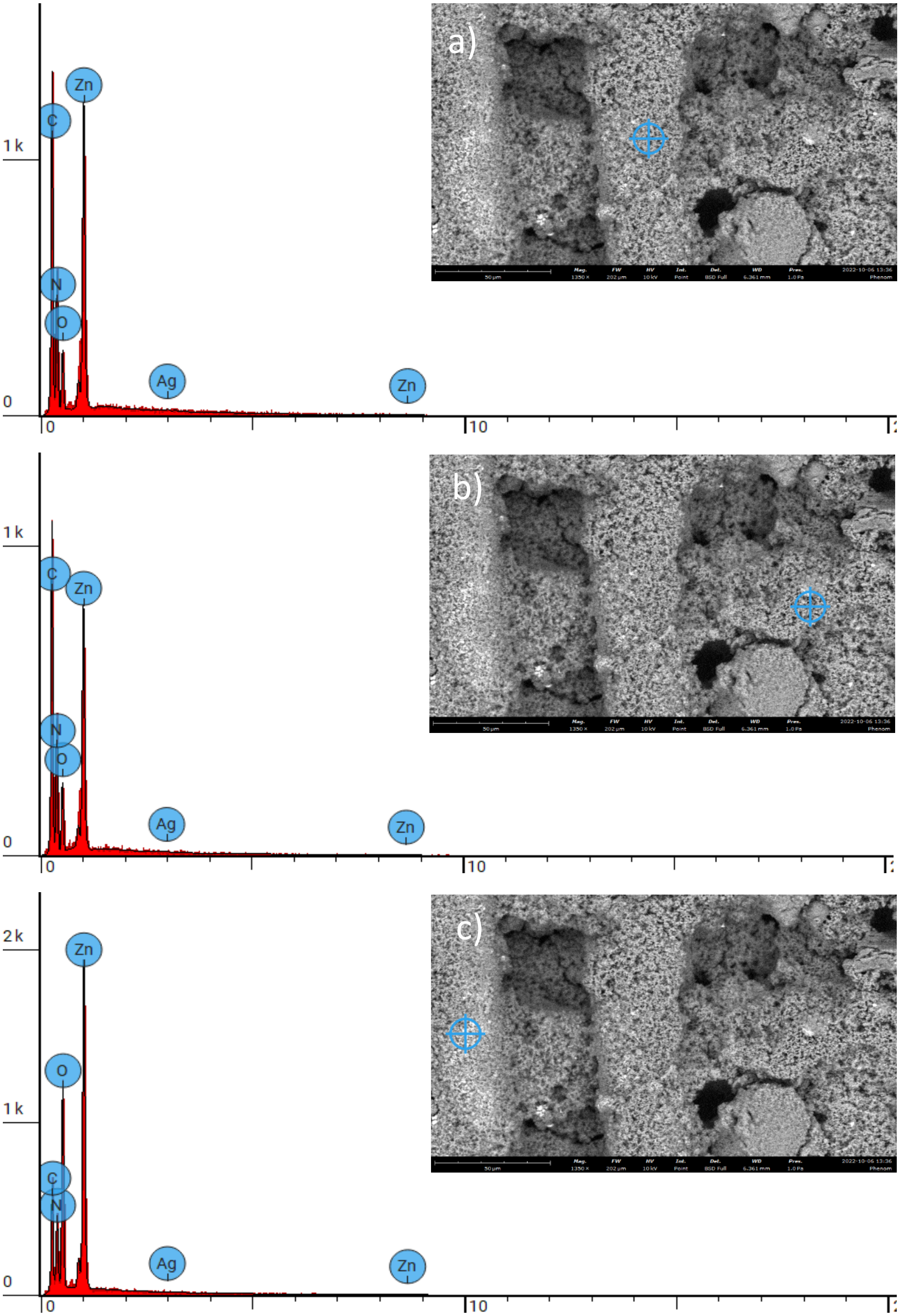

Figure S5. SEM images and EDS spectra of three spots (a-c) on Ag/ZIF-8 coated SSM.

Table S1. A summary of elemental composition of Ag/ZIF-8 based on the EDS mapping.

	Element Number	Element Symbol	Element Name	Atomic Conc.	Weight Conc.
	6	C	Carbon	34.754	26.200
	7	N	Nitrogen	48.215	42.400
	8	O	Oxygen	12.546	12.600
	30	Zn	Zinc	4.337	17.800
	47	Ag	Silver	0.148	1.000

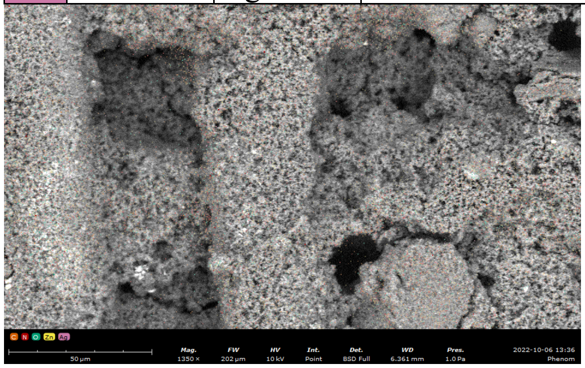

2. **Water remediation testing.**

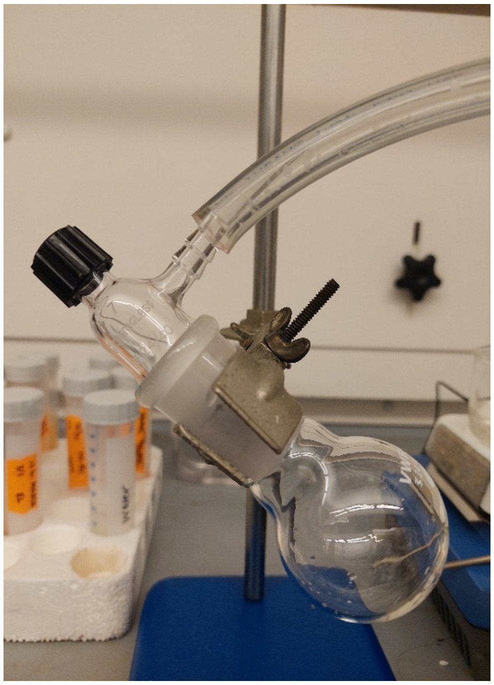

Figure S6. Optical image of the filtration system: stainless-steel mesh (SSM) membrane was fixed inside the black cap; the tubing was connected to in-house vacuum; rhodamine B solution was pushed by a syringe (not shown) to go through the membrane when the vacuum was kept on.

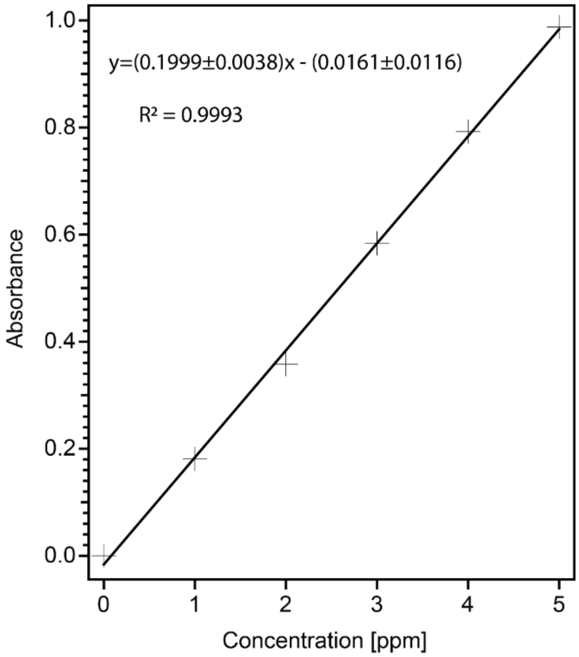

Figure S7. Calibration curve of rhodamine B measured using a UV-Vis spectroscopy at wavelength of 561 nm.
